# School Distribution as Keep-Up Strategy to Maintain Universal Coverage of Long-Lasting Insecticidal Nets: Implementation and Results of a Program in Southern Tanzania

**DOI:** 10.9745/GHSP-D-16-00040

**Published:** 2016-06-20

**Authors:** Shabbir Lalji, Jeremiah M Ngondi, Narjis G Thawer, Autman Tembo, Renata Mandike, Ally Mohamed, Frank Chacky, Charles D Mwalimu, George Greer, Naomi Kaspar, Karen Kramer, Bertha Mlay, Kheri Issa, Jane Lweikiza, Anold Mutafungwa, Mary Nzowa, Ritha A Willilo, Waziri Nyoni, David Dadi, Mahdi M Ramsan, Richard Reithinger, Stephen M Magesa

**Affiliations:** a RTI International, Dar es Salaam, Tanzania; b Ministry of Health and Social Welfare, National Malaria Control Programme, Dar es Salaam, Tanzania; c U.S. President’s Malaria Initiative/U.S. Agency for International Development, Dar es Salaam, Tanzania; d Swiss Tropical and Public Health Institute, Dar es Salaam, Tanzania; e Tanzania Red Cross Society, Dar es Salaam, Tanzania; f Johns Hopkins Center for Communication Programs, Dar es Salaam, Tanzania; g RTI International, Washington, DC, USA

## Abstract

Tanzania successfully scaled up coverage of long-lasting insecticidal nets (LLINs) through mass campaigns. To sustain these gains, a school-based approach was piloted in the country’s Southern Zone starting in 2013, called the School Net Program 1 (SNP1). We report on the design, implementation, monitoring, and outputs of the second round (SNP2) undertaken in 2014. SNP2 was conducted in all schools in Lindi, Mtwara, and Ruvuma regions, targeting students in primary (Standards 1, 3, 5, and 7) and secondary (Forms 2 and 4) schools and all teachers. In Lindi region, 2 additional classes (Standards 2 and 4) were targeted. LLIN distribution data were managed using an Android software application called SchoolNet. SNP2 included 2,337 schools, 473,700 students, and 25,269 teachers. A total of 5,070 people were trained in LLIN distribution (487 trainers and 4,583 distributors), and 4,392 (434 ward and 3,958 village) community change agents undertook sensitization and mobilization. A total of 507,775 LLINs were distributed to schools, with 464,510 (97.9% of those registered) students and 24,206 (95.8% of those registered) school teachers receiving LLINs. LLIN ownership and use is expected to have increased, potentially further reducing the burden of malaria in the Southern Zone of Tanzania.

## Abstract (precis)

A school-based net distribution program, piloted in the Southern Zone of Tanzania to sustain ≥80% universal net coverage previously attained through mass campaigns, successfully issued nets to nearly all eligible students and teachers. Keys to success included:

- Effective collaboration between the Ministry of Health, local government, and implementing partners
- Social mobilization to sensitize the community about the importance of net use
- Development of a mobile application to facilitate data collection and analysis

## BACKGROUND

Mass distribution of insecticide-treated nets, specifically long-lasting insecticidal nets (LLINs), is an effective vector control intervention to prevent malaria^1^^,^^2^ and has been associated with large reductions in malaria morbidity and mortality.^2^ The use of LLINs in sub-Saharan Africa has increased in the past decade to reach 80% universal coverage (defined as 1 LLIN per 2 people in a household).^3^^–^^5^ Since 2000, the Tanzania National Malaria Control Programme (NMCP) has led the National Insecticide Treated Nets (NATNETS) Programme to scale up LLIN distribution and use in Tanzania. NATNETS introduced the Tanzania National Voucher Scheme (TNVS) to provide pregnant women and infants with LLINs at highly discounted prices.^6^^,^^7^ The TNVS was successful in distributing LLINs to pregnant women and infants; however, not all eligible people were accessing health facilities to benefit from the program and not all people attending health facilities redeemed their vouchers.^8^^–^^10^

Not all eligible people in Tanzania received LLINs through the voucher program, either because they did not attend health facilities or did not redeem vouchers.

To ensure that the entire population (including those not covered by the TNVS) would have access to LLINs, 2 LLIN mass campaigns were implemented: (1) the children under 5 years of age catch-up campaign (U5CC) in 2009 and (2) the universal coverage campaign in 2011.^11^ Between 2009 and 2011, these campaigns distributed approximately 27 million LLINs, in addition to more than 3 million nets delivered through the TNVS.^11^^–^^13^ The TNVS, U5CC, and universal coverage campaign were successful in dramatically increasing LLIN ownership in Tanzania. Household net ownership increased from 38% in 2007 to more than 85% in 2011, and use of LLINs increased from 25% in 2007 to 63% in 2011.^14^

To maintain at least 80% universal coverage of LLINs, the Ministry of Health and Social Welfare developed a keep-up strategy in schools to complement the TNVS. Keep-up strategies focus on long-term, continuous distribution through channels such as schools, health facilities, community initiatives, and the private sector; catch-up strategies are periodic mass distribution campaigns. NATNETS assessed all possible keep-up options and determined that a combined approach including the TNVS and school-based distribution, involving both push and pull systems, was the most cost-effective and efficient keep-up strategy for Tanzania.^12^^,^^15^^–^^17^ Because school enrollment in Tanzania is high and teachers can easily facilitate and manage the delivery of LLINs, school-based distribution of LLINs can be an effective strategy to reach many households.^18^

The Ministry developed a keep-up strategy in schools to complement the voucher scheme.

In 2013, the NMCP and partners piloted the first round of the School Net Program (SNP1) to distribute LLINs in the Southern Zone of Tanzania (Lindi, Mtwara, and Ruvuma regions). Each student in Standards 1, 3, 5, and 7 of primary schools, and Forms 2 and 4 of secondary schools, received an LLIN to take home. A total of 421,285 students (83% of those eligible) received LLINs.^19^ The assumption behind this strategy was that as the children moved through the school system, they would bring home a new LLIN every 2 years, which would be redistributed within the household or community. The pilot program demonstrated that it was feasible to rapidly and equitably distribute large quantities of LLINs through schools to the community. Household ownership of at least 1 LLIN increased by almost 17% compared with an area not covered under SNP1.^20^ Use of LLINs among older children and adolescents also increased.

We describe here the design, implementation, monitoring, and outputs of the second round of the School Net Program (SNP2), conducted in 2014.

## METHODS

### Study Setting

**FIGURE 1:**
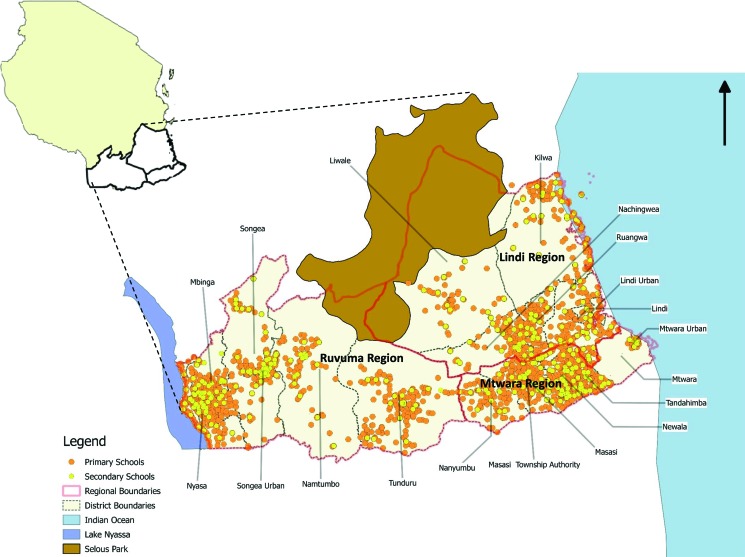
Locations of Schools Implementing the Second Round of the School Net Program (SNP2) in the Southern Zone of Tanzania, 2014

Figure 1 shows a map of Tanzania with the locations of schools in the Southern Zone (Lindi, Mtwara, and Ruvuma regions) and 19 districts where SNP2 was implemented. In 2012, the total populations by region were 864,652 in Lindi; 1,270,854 in Mtwara; and 1,376,891 in Ruvuma.^21^ In 2011, the prevalence of malaria among children under 5 years of age ranged from 12% in Ruvuma and 17% in Mtwara to 26% in Lindi.^14^ In 2010, the gross enrollment ratio, which refers to the total number of students of any age enrolled in school expressed as a percentage of the official school-age population, was 94.8%, 102.5%, and 106.6% for primary schools and 11.1%, 31.2%, and 33.6% for secondary schools in Lindi, Mtwara, and Ruvuma, respectively.^22^ The national average gross enrollment ratio was estimated at 99.0% and 31.8% for primary and secondary schools, respectively.

### Study Design

SNP2 aimed to increase access to LLINs and maintain at least 80% coverage in the 3 regions by distributing LLINs free of charge to primary and secondary school students and teachers. Teachers were included as beneficiaries in SNP2 because there was an excess supply of LLINs from SNP1. In Lindi, an additional 2 grades (Standards 2 and 4) were targeted for LLIN distribution to reduce the expected remaining stock. As in SNP1, each student from Standards 1, 3, 5, and 7 and Forms 2 and 4 in Mtwara and Ruvuma received 1 LLIN.

### Partnership, Coordination, and Planning

An SNP task force, chaired by the NMCP, was created to oversee the implementation of SNP2 (Box). The task force played a key role in the planning, coordination, and implementation of SNP2. It ensured that LLIN distribution aligned with the NMCP’s LLIN strategy and that partners were involved in key implementation decisions. It also took into account the recommendations, experiences, and lessons learned from SNP1. Advocacy meetings were held before LLIN distribution at national, regional, and district levels. Subcommittees were formed to plan specific activities, including LLIN quantification and logistics, training, social mobilization, and monitoring and evaluation (M&E).

**BOX. Members of the School Net Program 2 (SNP2) Task Force**

Members of the SNP2 task force included representatives from the:

- National Malaria Control Programme
- Ministry of Health and Social Welfare School Health Program
- Ministry of Education and Vocational Training
- Prime Minister’s Office Regional Authority and Local Government
- Swiss Tropical and Public Health Institute’s NETCELL project
- U.S. President’s Malaria Initiative
- World Health Organization
- Johns Hopkins Center for Communication Programs
- U.S. Peace Corps
- Mennonite Economic Development Associates
- RTI International
- Tanzania Red Cross Society
- Population Services International
- John Snow, Inc.

### LLIN Quantification

Before the SNP1 pilot, we used the NetCALC Planning Tool (www.vector-works.org/resources/netcalc-planning-tool) to estimate the quantity of nets required to maintain at least 80% universal coverage in the Southern Zone of Tanzania.^12^ For SNP2, the number of students attending each school was estimated using SNP1 registration records. We also obtained data from the district education department on the number of students and teachers in each school. Data were compared and verified to determine if there were any discrepancies. Data for eligible classes and teachers in each school were used to estimate the number of LLINs to deliver to each school. A contingency amount of 3% of the overall number of estimated LLINs for each school was also included.

### Training of Trainers and Implementers

The SNP task force training subcommittee was responsible for reviewing and revising all required training manuals. The strategy for training in SNP2 was a cascade approach, beginning with training of trainers (TOT) at the district level, who then trained implementers (teachers) at the ward level. This was a shift away from a more centralized model used in SNP1, which required a substantial period of time, as one team of trainers moved from one district and region to another to conduct trainings. The training period for SNP2 was also reduced from a 2-day session to a half-day session at both the district and ward levels. The TOT targeted district malaria focal persons, district school health coordinators for health and education, and the ward education coordinators. The training of implementers was at the ward level, whereby head teachers and school health teachers from all schools within a ward attended the training. The ward education coordinators were the principal trainers who were supervised by national, regional, and district-based staff. Training focused on the use of LLINs, continuous distribution of LLINs through schools, monitoring, and reporting. Participants learned a systematic approach to managing the LLIN distribution process, including handling data collection forms, enumerating students, recording LLIN issuance data, summarizing data, and submitting data.

### Social Mobilization

The IEC/BCC subcommittee of the SNP task force planned advocacy and sensitization meetings; reviewed information, education, and communication (IEC) and behavior change communication (BCC) materials; and trained community change agents and volunteers. The task force distributed IEC materials, and aired radio spots and programs on local radio stations in Ruvuma, Lindi, and Mtwara regions to sensitize the community on the importance of LLIN ownership and use. IEC materials and radio spots focused on the community aspect of LLIN distribution. Spots provided details on the importance of using LLINs, emphasizing that students who received LLINs served as conduits for delivering them to the community.

### Development of Tools and Manuals

The M&E subcommittee of the SNP task force was set up to revise data collection tools and training manuals. The M&E team developed registration and issuance booklets as well as summary booklets to capture data at the school level during LLIN issuance. They used the summary booklets, filled in by the school principal, to compare the number of LLINs issued against the number of students in the registration and issuance booklets, filled in by class teachers.

### Implementation of the SchoolNet Application

We developed a database application called SchoolNet to facilitate the collection and management of data from SNP2 schools. The SchoolNet app runs on Android tablets. We trained data clerks to use the SchoolNet app and developed an operative manual for guidance. Data clerks were responsible for entering data from the respective schools onto the tablets. Data were transmitted in real time through the local mobile phone network to a central database where the M&E team cleaned and analyzed the data in a timely manner for programmatic decision making. This approach to data management reduced the time-lag between LLIN issuance and data reporting, which enabled prompt analysis while maintaining high standards of data quality.

A tablet-based application called SchoolNet enabled real-time data collection from schools on LLIN issuance.

### Delivery of LLINs

The logistics subcommittee of the SNP task force was responsible for reviewing the transport and logistics protocols, streamlining distribution procedures, and developing a program and timeline to distribute LLINs from Dar es Salaam to all SNP2 schools. The bulk transportation and distribution of LLINs to schools was led by the Tanzania Red Cross Society (TRCS), a humanitarian organization with expertise in logistics and supply chain management. Moving the LLINs from Dar es Salaam to schools involved geographical reconnaissance, transportation planning, rebundling, storage, and transport to the schools through the districts. TRCS transported LLINs from the central-level medical store department warehouse to the district level, rebundled them according to the quantification data, and stored them in warehouses until distribution to respective SNP2 schools.

#### Geographical Reconnaissance

Logistics teams were dispatched to all districts to collect data regarding storage facilities, availability of council and private transports, and maps to design distribution routes. The geographic reconnaissance data were critical to developing a comprehensive logistics and transportation schedule, as it took into account geographical opportunities and challenges, such as poor road infrastructure in hard-to-reach areas.

#### Rebundling and Transport of LLINs

Rebundling LLINs involved grouping them into bales according to the needs of each school in the district, based on quantification data gathered during the SNP2 planning phase. TRCS packed LLINs in smaller volumes and labeled each with the quantity of LLINs and the respective school name. Labeled bundles were loaded onto smaller vehicles for ease of access on rural roads to ensure that they reached the targeted schools. To properly account for the LLIN inventory, SNP2 used delivery notes, warehouse journals, and bin cards to document and track net movement at every point of delivery. We conducted an independent procedural audit to track the LLINs from the central warehouse in Dar es Salaam, to a sample of 3 districts warehouses and to a sample of 27 schools. The audit reported that the logistics chain was effective in delivering LLINs and that there were no losses.

### LLIN Issuance to Schools and Definition of LLIN Coverage

LLIN delivery and issuance to all primary and secondary schools took place over a 3-day period on regular school days. Class teachers organized preregistered students by their respective classes. Each student and teacher receiving an LLIN were required to sign against his or her name in the registration and issuance booklets as proof of having received an LLIN. Supervision and documentation focused on the integrity of school data; adherence to procedures for distribution; and feedback on successes, challenges, and areas for improvement related to the LLIN distribution and issuing. We defined LLIN coverage as the proportion of eligible students and teachers who received LLINs.

### Data Collection, Management, and Analysis

With technical assistance and supervision from the M&E team, data clerks entered data from hard copy training reports, inventory booklets, attendance registers, and school registers in central databases via the SchoolNet app. All information was subjected to periodic data quality checks through regular supervision and review of documents. The head teacher or school principal filled in summary booklets for each school, which the M&E team used to compare the number of LLINs issued against the number of students registered in each class. The issuance forms also captured the number of LLINs that schools received from the district warehouses, thus enabling the schools to keep track of remaining stocks.

Issuance forms captured the number of LLINs that schools received, enabling them to track remaining stock.

Data from each school were submitted to ward education coordinators for verification and subsequently sent to the district education officers. The M&E team then verified and cleaned the data. The M&E team visited all district offices to supervise the data collection process and to verify the quality, tools, and methodology used. To verify the data entered onto the tablets by the data clerks, the M&E team cross-checked them with hard copies of the issuance forms and summary booklets. All hard copy records were reviewed for accuracy and consistency, and filed in a central repository. Naming conventions were assigned for easy storage and retrieval of the records on hand. Relevant data were extracted from the SchoolNet app database and exported to a Microsoft Excel spreadsheet for analysis.

The M&E team used Stata 12.0 to conduct data analysis. The team used descriptive statistics to investigate frequency distributions and proportions, and box plots to investigate the distributions of number of students eligible for SNP by class and region.

### Ethical Consideration

SNP2 was undertaken as part of public health programming, and therefore ethical clearance was not required a priori. The National Health Research Ethics Subcommittee granted written permission to publish these data.

## RESULTS

### Training of Trainers and Implementers

Table 1 shows the number of people trained as trainers and implementers (teachers) in the targeted 19 districts in 3 regions, by gender. Overall, 487 trainers (78.1% male) and 4,583 teachers as implementers (66.8% male) were trained.

**TABLE 1:** Number of Trainers and Implementers Trained for SNP2, by District and Gender, 2014

		Trainers		Implementers (Teachers)	
Region	District	Total	% Male	Total	% Male
Lindi	Kilwa District Council	27	96.3%	250	82.8%
	Lindi District Council	25	80.0%	223	65.9%
	Lindi Municipal Council	11	54.5%	82	56.1%
	Liwale District Council	23	82.6%	139	74.8%
	Nachingwea District Council	34	70.6%	258	65.5%
	Ruangwa District Council	23	69.6%	192	65.1%
Subtotal		143	77.6%	1,144	69.8%
Mtwara	Masasi District Council	26	76.9%	293	65.5%
	Masasi Town Council	14	64.3%	90	47.8%
	Mtwara District Council	31	80.6%	298	73.8%
	Mtwara Municipal Council	18	83.3%	104	58.7%
	Nanyumbu District Council	17	76.5%	200	73.5%
	Newala District Council	31	74.2%	291	69.1%
	Tandahimba District Council	34	88.2%	289	72.0%
Subtotal		171	78.9%	1,565	68.5%
Ruvuma	Mbinga District Council	44	75.0%	552	59.8%
	Namtumbo District Council	23	95.7%	283	63.6%
	Nyasa District Council	21	90.5%	226	78.8%
	Songea District Council	21	76.2%	245	60.4%
	Songea Municipal Council	23	78.3%	230	49.6%
	Tunduru District Council	41	82.9%	338	71.0%
Subtotal		173	82.1%	1,874	63.5%
**Grand Total**		**487**	**79.7%**	**4,583**	**66.8%**

Abbreviation: SNP2, School Net Program – Round 2.

### Social Mobilization

Table 2 shows the number of IEC and BCC materials distributed during SNP2 by region. In addition, 2,600 radio spots and 23 *PataPata* children’s radio programs were broadcast through local radio stations, including Jogoo FM, Pride FM, Newala FM, and the Tanzania Broadcasting Corporation. A total of 4,392 community change agents (434 at the ward level and 3,958 at the village level) delivered 1,500 sensitization and mobilization sessions, reaching 42,626 people.

Comprehensive social mobilization activities complemented LLIN distribution, including IEC and BCC materials, radio spots, and sensitization sessions.

**TABLE 2:** Number of IEC Materials Distributed by SNP2, by District, 2014

Type of IEC Material	Mtwara	Lindi	Ruvuma	Total
Community posters (*Tushirikiane Kutokomeza Malaria*)	4,878	3,738	3,000	11,616
School posters (*Mwanafunzi Shiriki*)	3,975	3,025	3,700	10,700
Multiplication tables	64,125	45,600	70,775	180,500
Patapata posters	3,225	2,400	3,725	9,350
Comic brochures	166,667	166,667	166,666	500,000
Patapata notebooks	5,285	10,220	9,905	25,410
Patapata notebooks for radio station	5	5	5	15
Teacher cue cards	1,935	1,440	2,235	5,610
Community change agents cue cards (*Dondoo za Majadiliano*)	302	282	284	868

Abbreviations: IEC, information, education, and communication; SNP2, School Net Program – Round 2.

### Participation in the Program

**FIGURE 2:**
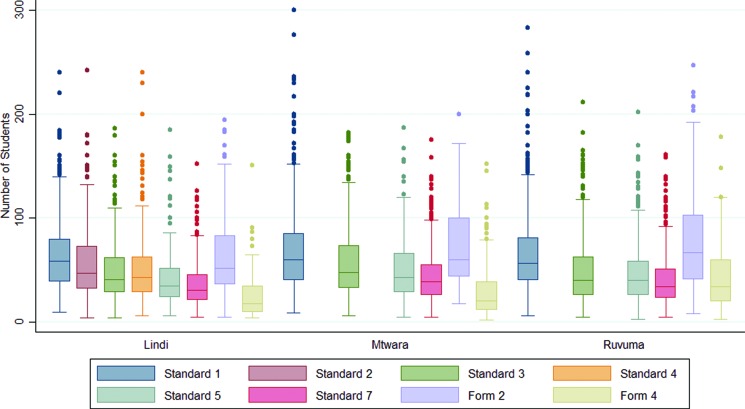
Distribution of Registered Students Eligible to Receive a Long-Lasting Insecticidal Net, by Class and Region

All 2,337 schools participated in SNP2-targeted districts, the majority of which were primary schools (Table 3). The number of schools varied within each district, ranging from 41 in Lindi Municipal Council to 269 in Mbinga District Council. A total of 473,700 students were registered in SNP2 schools and were eligible to participate in the distribution. Primary school students in Standards 1, 3, 5, and 7 comprised 89.3% of the total number of eligible students. Figure 2 shows the distribution of registered students by region for each grade eligible to receive an LLIN through SNP2. The number of registered students declined in each subsequent grade, with a median of 59 (interquartile range [IQR], 40–83) students in Standard 1 compared with a median of 34 (IQR, 23–51) students in Standard 7. In secondary schools, the median (IQR) students in Form 2 and Form 4 were 60 (40–97) and 25 (14–49), respectively (Figure 2). A total of 25,269 teachers were registered in all schools (Table 3).

**TABLE 3:** Schools, Eligible Students, and Teachers Participating in SNP2, by District, 2014

Region	District	No. of Schools			No. of Students^a^									No. of Teachers
		Primary	Secondary	Total	Std. 1	Std. 2	Std. 3	Std. 4	Std. 5	Std. 7	Form 2	Form 4	Total	
Lindi	Kilwa District Council	108	28	136	7,483	6,283	5,925	5,631	4,604	4,255	1,448	547	36,176	1,226
	Lindi District Council	114	27	141	7,364	5,972	4,965	5,420	3,987	3,428	1,428	574	33,138	1,218
	Lindi Municipal Council	32	9	41	2,421	1,937	1,853	1,766	1,567	1,358	843	400	12,145	619
	Liwale District Council	54	16	70	4,225	3,220	2,982	3,204	2,582	2,035	1,233	309	19,790	654
	Nachingwea District Council	103	26	129	6,302	5,890	4,769	4,299	3,860	3,192	1,755	428	30,495	1,222
	Ruangwa District Council	82	15	97	4,301	3,829	3,144	3,526	2,468	2,119	873	313	20,573	886
Subtotal		493	121	614	32,096	27,131	23,638	23,846	19,068	16,387	7,580	2,571	152,317	5,825
Mtwara	Masasi District Council	126	23	149	8,645		6,838		6,024	5,439	1,863	1,156	29,965	1,588
	Masasi Town Council	37	8	45	3,130		2,214		1,776	2,003	888	410	10,421	714
	Mtwara District Council	127	24	151	8,253		7,201		5,879	5,108	1,304	457	28,202	1,360
	Mtwara Municipal Council	33	21	54	3,232		2,641		2,394	2,096	2,079	915	13,357	1,112
	Nanyumbu District Council	87	11	98	6,804		4,532		3,577	2,631	1,053	152	18,749	885
	Newala District Council	118	28	146	6,175		4,963		5,207	4,087	1,740	534	22,706	1,419
	Tandahimba District Council	115	28	143	7,852		6,964		5,998	5,007	1,630	506	27,957	1,485
Subtotal		643	143	786	44,091		35,353		30,855	26,371	10,557	4,130	151,357	8,563
Ruvuma	Mbinga District Council	227	52	279	11,583		8,815		9,078	7,833	3,634	1,745	42,688	2,601
	Namtumbo District Council	108	29	137	7,505		5,980		5,131	4,526	2,060	1,077	26,279	1,561
	Nyasa District Council	97	18	115	5,721		4,594		4,235	3,197	1,116	534	19,397	964
	Songea District Council	95	27	122	6,086		5,168		4,393	3,715	1,776	820	21,958	1,647
	Songea Municipal Council	78	37	115	7,547		5,740		5,234	4,557	3,664	1,985	28,727	2,365
	Tunduru District Council	146	23	169	10,887		5,889		5,902	4,944	2,370	985	30,977	1,743
Subtotal		751	186	937	49,329		36,186		33,973	28,772	14,620	7,146	170,026	10,881
**Grand Total**		**1,887**	**450**	**2,337**	**125,516**	**27,131**	**95,177**	**23,846**	**83,896**	**71,530**	**32,757**	**13,847**	**473,700**	**25,269**

Abbreviation: SNP2, School Net Program – Round 2.

a Standard 2 and Standard 4 students were eligible for SNP2 in Lindi region only.

### LLINs Issued to Schools and LLIN Coverage

Schools received a total of 507,775 LLINs, of which 91.5% and 4.8% were issued to students and teachers, respectively (Table 4). After distribution, 3.7% of LLINs remained, ranging from 1.7% in Ruvuma to 5.4% in Lindi.

**TABLE 4:** LLINs Issued to Teachers and Students by SNP2, by District, 2014

Region	District	No. of LLINs:					Percentage of:		
		Received by School	Issued to Students	Issued to Teachers	Total Issued	Remaining After Issuing	Registered Students Issued LLINs	Registered Teachers Issued LLINs	LLINs Remaining
Lindi	Kilwa District Council	35,575	35,702	1,206	36,781	1,903	98.3%	98.4%	4.6%
	Lindi District Council	32,425	32,451	1,195	33,620	4,195	97.9%	98.1%	11.0%
	Lindi Municipal Council	11,853	11,901	619	12,472	200	97.7%	100.0%	1.2%
	Liwale District Council	19,730	19,807	653	20,383	461	99.7%	99.8%	1.8%
	Nachingwea District Council	29,555	29,660	1,112	30,667	421	96.7%	91.0%	1.0%
	Ruangwa District Council	20,194	20,194	868	21,062	1,993	98.2%	98.0%	8.6%
Subtotal		149,332	149,715	5,653	154,985	9,173	98.0%	97.0%	5.4%
Mtwara	Masasi District Council	28,932	28,932	1,501	30,433	2,740	96.5%	94.5%	8.3%
	Masasi District Council	10,095	10,095	664	10,759	175	96.6%	93.0%	1.6%
	Mtwara District Council	26,896	26,896	1,266	28,162	719	95.3%	93.1%	2.5%
	Mtwara Municipal Council	13,244	13,244	1,050	14,294	100	98.8%	94.4%	0.7%
	Nanyumbu District Council	18,681	18,681	878	19,559	1,384	99.6%	99.2%	6.6%
	Newala District Council	22,314	22,314	1,322	23,636	1,185	98.0%	93.2%	4.8%
	Tandahimba District Council	26,310	26,310	1,317	27,627	508	93.8%	88.7%	1.8%
Subtotal		146,472	146,472	7,998	154,470	6,811	96.6%	93.4%	4.2%
Ruvuma	Mbinga District Council	42,380	42,380	2,550	44,930	670	99.2%	98.0%	1.5%
	Namtumbo District Council	26,133	26,133	1,483	27,616	393	99.2%	95.0%	1.4%
	Nyasa District Council	19,281	19,281	943	20,224	98	99.3%	97.8%	0.5%
	Songea District Council	21,525	21,525	1,584	23,109	607	97.9%	96.2%	2.6%
	Songea Municipal Council	28,689	28,689	2,365	31,054	1,074	99.9%	100.0%	3.3%
	Tunduru District Council	30,698	30,698	1,630	32,328	233	98.8%	93.5%	0.7%
Subtotal		168,706	168,706	10,555	179,261	3,075	99.1%	97.0%	1.7%
**Grand Total**		**507,775**	**464,510**	**24,206**	**488,716**	**19,059**	**97.9%**	**95.8%**	**3.7%**

Abbreviations: LLIN, long-lasting insecticidal net; SNP2, School Net Program – Round 2.

**FIGURE 3:**
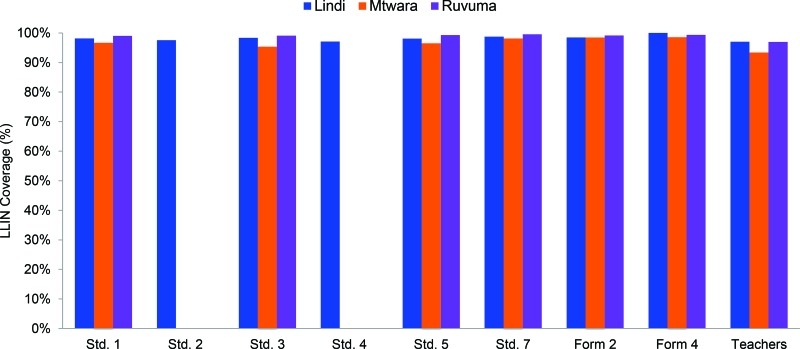
Percentage of Eligible Students and Teachers Receiving a Long-Lasting Insecticidal Net, by Class and Region

**FIGURE 4:**
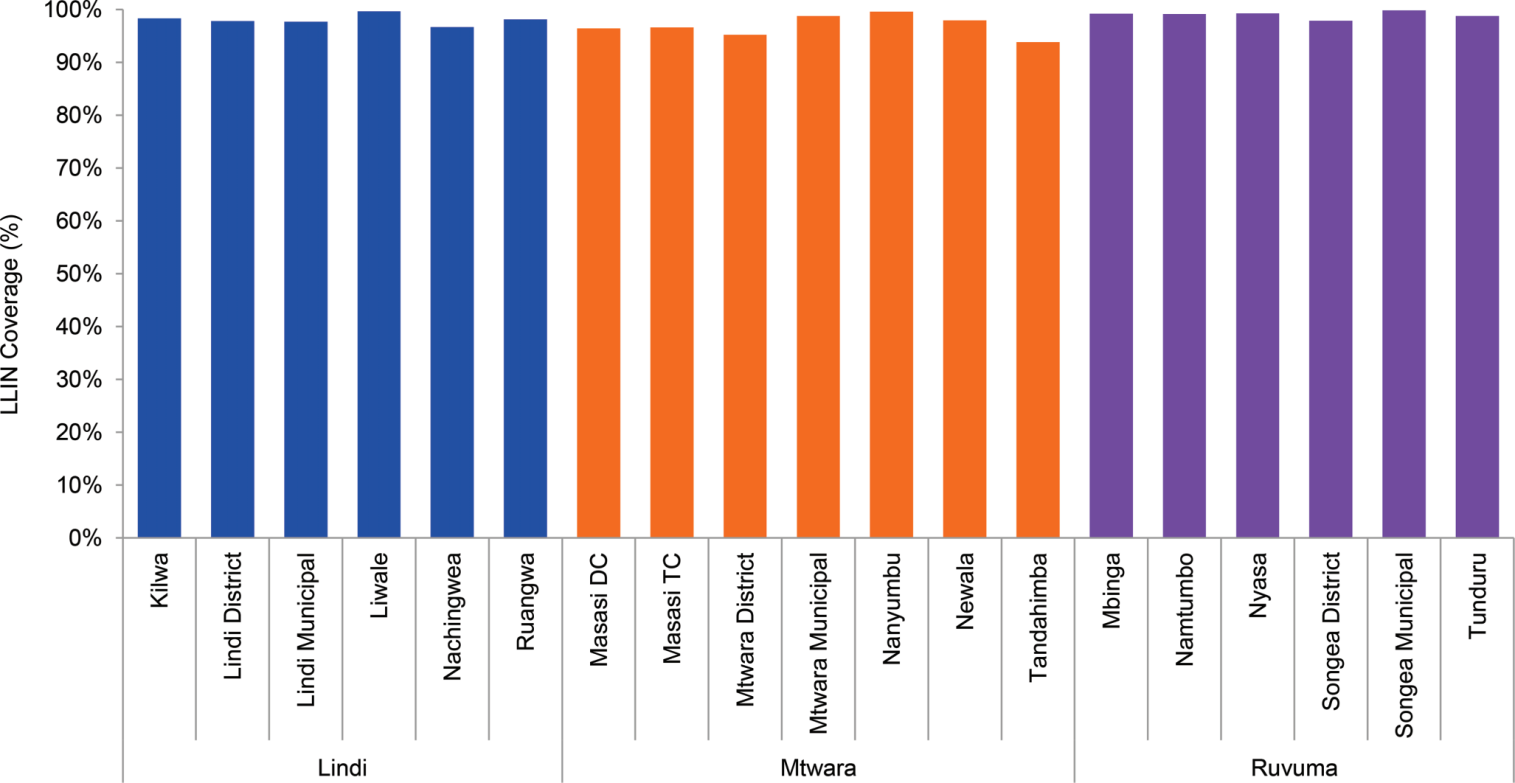
Percentage of Eligible Students and Teachers Receiving a Long-Lasting Insecticidal Net, by District and Region

Figure 3 shows the distribution of LLIN coverage by grade and region, and Table 4 and Figure 4 shows coverage by district. Overall, 97.9% of students and 95.8% of teachers received LLINs in the 3 regions. Mtwara Region had the lowest LLIN coverage for both teachers and students, at 96.6% and 94.5%, respectively. Across all districts, the percentage of teachers and students that received LLINs was consistently above 90% (Figure 3 and Figure 4).

Overall, 98% of eligible students and 96% of eligible teachers received LLINs in the 3 regions.

## DISCUSSION

The school-based net distribution program, SNP2, successfully issued LLINs to 98% of eligible students and 96% of eligible teachers in the 3 targeted regions of Tanzania. Our findings are consistent with a similar pilot study in Ghana where LLINs were distributed to students in grades 2 and 6 of primary schools, teachers, and district office team members, and achieved nearly 100% LLIN coverage among eligible participants.^23^ Independent evaluations of SNP1 and SNP2 in Tanzania found that this school-based approach resulted in increased household ownership and improved usage of LLINs compared with control districts with no school-based delivery mechanism.^24^^,^^25^ Following SNP2 implementation, household ownership of LLINs was 84% in Mtwara, 84% in Lindi, and 75% in Ruvuma, and universal coverage of LLINs (households with 1 net for every 2 people) was estimated at 70% in Mtwara, 65% in Lindi, and 56% in Ruvuma. In comparison, universal coverage of LLINs in non-SNP2 control districts in Lake Zone was lower: 33% in Chato District and 46% in Sengerema District.^25^ Despite 2 rounds of SNP, coverage of LLINs across the 3 SNP target regions was still below the universal coverage target of 80%. This is largely attributed to the fact that SNP started in 2013, 2 years after the 2011 mass LLIN distribution campaign. Based on NetCALC models, LLIN coverage would have declined by 50% to 60% between 2011 and 2013.^12^^,^^26^ To maintain universal coverage, we recommend that continuous distribution through SNP should be implemented within 9 to 12 months of any mass LLIN distribution campaign.^26^

We recommend implementing continuous LLIN distribution through schools within 9 to 12 months of any mass distribution campaign.

Compared with SNP1, SNP2 achieved higher LLIN coverage of students (98% versus 83%), distributed 16% more nets, and had fewer LLINs remaining after distribution (4% versus 17%) due to a change in the program design to include teachers in all regions and additional grades in Lindi region. SNP2 was built on effective collaboration and teamwork between the Ministry of Health and Social Welfare, local government, and implementing partners. Specific roles and responsibilities were assigned to working groups to implement elements of the SNP framework—a crucial ingredient for the success of SNP2 activities, as demonstrated in previous campaigns conducted in Tanzania.^8^^,^^11^ Some adjustments were made to the pilot SNP1 approach, which also contributed to SNP2’s success. These included (1) providing training to the trainers and implementers; (2) implementing comprehensive social mobilization activities^12^^,^^25^; (3) introducing the SchoolNet app, enabling real-time data entry into a central database, more effective data management, feedback on data quality, and easier and more timely data analysis and dissemination; and (4) timing the LLIN distribution in August (i.e., during the dry season) to ensure easier access to all schools for LLIN delivery.

Slight differences between school enrollment data provided by districts (used for quantification) and student registration at schools (recorded in real time during issuing) may have resulted in a deficit or oversupply of LLINs in a small proportion of schools. Such deficits were resolved through redistribution of excess LLINs from neighboring schools. We therefore recommend that head teachers should provide school enrollment data for net quantification. Also, based on feedback from participants, the half-day training for trainers and implementers was not adequate to cover all necessary SNP2 details; trainings should be extended to 1 day in future rounds of the SNP. We also recommend that the training of key implementers (teachers) should be coordinated by the Ministry of Education and Vocational Training, who should be more engaged in SNP. Finally, models have shown that 35% of households are potentially not reached by either the TNVS or SNP.^24^ Registration data suggest that as students progress to higher grades, dropout rates increase, meaning that in households where all children drop out of school, they will not be eligible to receive LLINs. Thus, while it is expected that some households not eligible for LLINs via the TNVS or SNP could benefit from LLIN redistribution within households, further strategies are needed to ensure universal access to LLINs by the general population. The results of this program show that substantial proportions (89.3%) of students eligible for SNP were from primary schools; therefore, future school-based LLIN distribution should consider targeting primary school students only.

Future school-based LLIN distribution should consider targeting primary school students only.

## CONCLUSION

SNP2 was successful in reaching 98% of eligible students and 96% of eligible teachers in 3 regions in Tanzania. Effective partnerships, coordination, and teamwork facilitated the successful implementation of SNP2. LLIN ownership and use in the community is expected to increase and therefore reduce the burden of malaria in the 3 regions. This program can serve as a model for other countries who wish to implement a school-based approach as a keep-up strategy to maintain universal coverage of LLINs for malaria prevention and control.

## Notes

**Peer Reviewed**
